# Skateboarding’s Olympic journey: do the performance profiles of top athletes remain consistent?

**DOI:** 10.3389/fphys.2025.1548442

**Published:** 2025-03-19

**Authors:** Chloé Fouillot, Guillaume Saulière, Juliana Antero, Adrien Sedeaud, Jean-François Toussaint

**Affiliations:** ^1^ Institut de Recherche bio-Médicale et d’Épidémiologie de Sport, EA7329, INSEP, Université de Paris Cité, Paris, France; ^2^ Fédération Française de Roller et Skateboard, Bordeaux, France; ^3^ Centre d’Investigation en Médecine du Sport, Hôtel-Dieu, Assistance Publique, Hôpitaux de Paris, Paris, France

**Keywords:** potential estimation, youth development, young athletes, skateboarding, Olympics, performance trajectories, peak age, talent identification

## Abstract

**Introduction:**

Since its inclusion in the Tokyo 2020 Olympics, skateboarding has highlighted diverse age profiles among elite athletes. This study aims to characterize the relationship between age and performance in Street Skateboarding and examine how it has evolved with the sport’s growing professionalization.

**Methods:**

The dataset includes 2,822 performances by 498 women and 12,116 by 2,784 men from international street skateboarding competitions (2001–2024). Athlete number and gender ratios were compared between 2017 and 2024 vs 2001–2016 periods in order to assess the evolution of the competitive context, with analysis of new and returning athletes. Performance was quantified using a dynamic rank-order logit model (ROL). Moore’s exponential model and IMAP tracked the age-performance relationship, with adjusted R-squared for model fit. Peak performance ages, estimated performances, and AUC were analyzed for trends.

**Results:**

Since 2016, the number of international competitors has doubled for men (x2.13) and quadrupled for women (x4.02), narrowing the male-to-female participating ratio from 10.56 to 5.59. Records for new competitors have been consistently surpassed, particularly for women. Both Moore and IMAP models strongly explain the age-performance relationship (*R* ^2^: 0.84–0.94). According to Moore and IMAP models, peak performance ages are estimated at respectively 18.12 and 16.25 for women and 22.51 et 21.34 for men. For women, age of peak performance significantly evolved toward younger ages over time, with a sharper drop from 2021 to 2024. For men, both AUC and age at peak performance increased significantly, indicating a widening of the optimum performance age range.

**Discussion:**

The inclusion of skateboarding into the Olympic programs has expanded career opportunities, lowering peak performance age for women and broadening the optimal age range for men. This study is intended to help skateboarding stakeholders estimate athletes’ potential for future Olympiads and adapt development strategies. The age at peak performance trajectory for female athletes warrants special attention.

## 1 Introduction

Since its introduction to the Olympic Games in Tokyo 2020, the two female Street Skateboarding Olympic champions have won the title at ages 14. For the men, the 2022 Street Skateboarding world champion was nearly twice the age of the bronze medalist, while the same athlete won the first two Olympic Games in men Street skateboarding, highlighting the diverse age profiles and challenging the current characterization of high-performance skateboard athletes.

Moreover, the recent introduction of skateboarding to the Olympic Games raises the question of whether the characterization of its high-level athletes has changed over time. Entry into the closed circle of Olympism has led to an increase in research and publications in the chosen sports (Millet et al., 2021). In addition, a study carried out among practitioners and professional athletes of the extreme sports introduced at the last Olympic Games, as well as industry workers, found that the resulting gain in visibility may have increased their popularity, particularly among young people, and thus their participation (Renfree et al., 2021). This inclusion would also enable them to obtain better financial support from sports organizations, gain access to sponsorship contracts and consequently enable more athletes to make a living from their practice, especially women (Renfree et al., 2021). This professionalization of sports resulted in improvements in training, nutrition, recovery practices, and performance strategies, along with the development of new technologies aimed at enhancing performance (Berthelot et al., 2015; Norton and Olds, 2001). At the same time, the democratization of sports expanded the pool of participants, allowing for a broader selection of future top athletes (Berthelot et al., 2015; Norton and Olds, 2001). Consequently, both demographic expansion and professionalization possibly impact the age profiles of skateboard athletes.

Modeling the age-performance relationship appears to be an essential first step in this characterization, since it enables us to characterize numerous physical and physiological parameters inherent in sports performance as a function of time, such as strength (Mitchell et al., 2012), maximum oxygen consumption and respiratory volume (Stanojevic et al., 2008), pulmonary capillary volume (Aguilaniu et al., 2008), and cognitive performance (Park and Reuter-Lorenz, 2009). In 1975 (Moore, 1975), Moore was the first to propose methods explaining this relationship by summing two exponential laws: one increasing (performance progression phase), the other decreasing (regression phase) with, at the intersection of these two functions, a zone of optimum performance corresponding to peak performance (career peak).

More recently, a study (Berthelot et al., 2019) has used this model to create IMAP (Integrative Model of Age-Performance), the aim of which is to include biological aspects in the modeling of the asymmetrical relationship between age and performance, based on the biological unit represented by a cell.

The first of these models explains 91.7% of the variability in performance at the individual level and 98.5% of this variability from a species point of view (Berthelot et al., 2012), making it possible to define an age interval to which it would be preferable to belong, on the eve of a major event such as the Olympic Games, in order to maximize one’s chances of performance (Hollings et al., 2014). The application of this model to various events such as athletics (Berthelot et al., 2012; Schulz and Curnow, 1988; Marc et al., 2014), running (Schulz and Curnow, 1988; Knechtle et al., 2012a; Lepers and Cattagni, 2012), swimming (Dormehl et al., 2016; Allen et al., 2015), ultra-triathlon (Knechtle et al., 2012b; Knechtle et al., 2012c) or even sports with more difficult-to-measure performance indicators, such as tennis, baseball or golf (Schulz and Curnow, 1988; Guillaume et al., 2011), highlights these common trajectories: progression-peak-decline.

Additional methods have been explored for estimating the age of peak performance, including polynomial curve fitting, mixed models, rolling means, and various regression techniques (Allen and Hopkins, 2015). However, quadratic and other second-degree polynomial models, as previously employed (Lara et al., 2014; Simonton, 1988), tend to produce inaccurate estimates, as the relationship is consistently described as asymmetrical, with peak performance occurring relatively early in life (i.e., before mid-life) (Moore, 1975; Berthelot et al., 2019; Berthelot et al., 2012; Guillaume et al., 2011; Marck et al., 2017).

This study aims to model and characterize the relationship between age and performance in Street skateboarding by applying the previously outlined Moore and IMAP models accordingly to their proven relevance in fitting this relation discussed above and to examine its evolution since announcement of skateboarding’s inclusion in the Olympic program in 2016.

## 2 Materials and method

### 2.1 Description of the dataset

#### 2.1.1 Dataset composition

The dataset includes 2,822 performances achieved by 498 women competitors aged 8 to 39 and 12,116 performances performed by 2784 men competitors aged 8 to 47. Each performance corresponds to the final ranking of an athlete in a competition, among the 165 competitions held for female athletes and the 273 competitions held for male athletes between 2001 and the Paris 2024 Olympic Games.

#### 2.1.2 Ethical and regulatory considerations

These data were collected by Gracenote and provided to INSEP as part of their partnership. The collection of this data complies with the General Data Protection Regulations (GDPR) established by the European Union. The study was supervised and developed by the IRMES scientific committee. Ethical approval for the study protocol was obtained from the ethics panel of the Scientific, Medical, and Training Council (CSMF) at INSEP.

### 2.2 Skateboarding international competitive context evolution over time

For both men and women, the ratio of the total number of athletes participating in international competitions from 2001 to 2024, relative to the total number of athletes participating from 2001 to 2016, was calculated. The ratio of female athletes to male athletes for the period 2001–2016 was compared to the ratio for the entire period from 2001 to 2024. Number of new and former athletes competing on international events each year have been calculated to reflect the evolution over time of skateboarding international competitive context. The annual number of new competitors registered since 2016 was compared to the historical peak of new competitors recorded in the years prior to this period. Mean ± standard deviations of new athletes per year between 2001–2011 and 2012–2024 were computed and distributions were compared using Wilcoxon test.

### 2.3 Rating model

In skateboarding, the judges evaluate the athletes’ performances (run + best tricks) according to the following criteria: difficulty and variety of tricks performed, quality of execution, use of the skatepark and the various modules, flow and consistency, repetitions. Competition format, scoring ranges, and the calculation of total scores may vary depending on the organizers and changes in regulations over time. The scores awarded are meaningful primarily within the context of the competition in which the performance takes place. An identical performance may not necessarily receive the same score in a different competition. The rating system, by contrast, evaluates an athlete’s level relative to the competitive context in which their performance occurs. The score assigned to each athlete is updated with each competition based on their ranking and the strength of their competitors. Moreover, this system allows for standardized comparisons of competitor levels as soon as the start list for a new competition is available. For a given athlete, this score measures his level at each of his competitive appearances, considering his past performances as well as the competitive context in which the day’s performance takes place.

Rating scores were computed using the dynamic rank-order logit model (ROL) from Glickman and Hennesy (Glickman and Hennessy, 2015) which is especially adapted for multi-competitor games and sports as it takes into account the time-varying nature of athlete’s abilities.

This approach assumes that at each time period t competitor i has unknown ability parameter $\theta_{it}$ on which its ranking $Y_{i}$ depends:

$${Y_{i}\left|\theta \right.}_{it}∼Gumbel\left(\theta_{it}\right)$$

and which follows a stochastic process to account for temporal variation:

$$\theta_{it}∼N\left(\theta_{i,t-1},\tau^{2}\right)$$

with:

• Constraint that $\sum_{i=1}^{n}\theta_{it}=0$

• A prior distribution on initial abilities $\theta_{i1}∼N\left(0,\sigma_{1}^{2}\right)$ for all i .

The Gumbel distribution is particularly well-suited for Glickman and Hennessy’s dynamic ranking model for several reasons. It effectively captures the latent performance variability of individuals while preserving the relative order of competitors. Its natural connection to the logit model allows for an efficient formulation of ranking probabilities, which is crucial for modeling competitions. The Gumbel distribution’s simplicity enables concise mathematical expression and computational efficiency, making it easier to implement.

For fixed time period t, the probability of a ranking is a product of multinomial logit probabilities:

$$P\left(\left.Y_{1}>Y_{2}>\ldots >Y_{n}\right|\theta_{1},\theta_{2},\ldots ,\theta_{n}\right)=\prod_{i=1}^{m_{k}-1}\frac{e^{\theta_{i}}}{\sum_{l=i}^{m_{k}}e^{\theta_{l}}}$$

with $m_{k}$ the number of competitors during the competition k .

The logit model is used in rankings to model the probabilities of winning or losing. It transforms differences in ability into probabilities, which is useful for predicting outcomes. The logit model is simple to manipulate and suitable for classification and prediction. This makes it easy to model rankings efficiently.

As applying a full Bayesian treatment consisting of to obtain inferences for the model through Markov chain Monte Carlo (MCMC) simulation from the posterior distribution is computationally complex and time consuming, an alternative way consisting of estimate τ and $\sigma_{1}$ considering them as fixed and known and then successively updating the distribution of $\theta_{it}$ after competition date are observed to obtain the distribution for $\theta_{i,t+1}$ was implemented.

The Newton-Raphson algorithm aimed here at determining τ and $\sigma_{1}$ such that they maximize Spearman’s correlation coefficient between competitors’ ratings and their competition rankings was applied.

To do so, female and male datasets were each divided in two parts.

• A first one including 1/3 of the total number of competitions and allowing the initialization and construction of the rating from the final rankings.
• A second one for evaluating parameters $\tau_{k}$ et $\sigma_{1k}$ at each optimization iteration k .

After parameter optimization, the Spearman correlation between predicted and observed ratings is 0.563 for women and 0.603 for men.

### 2.4 Age-performance relationship and its evolution over time

Moore (Moore, 1975) modelled the relationship between age and performance as the sum of two exponential laws intersecting at peak performance.

$$P\left(t\right)=a\times \left(1-e^{-bt}\right)+c\times \left(1-e^{dt}\right)$$

with $P\left(t\right)$ the performance at age t and a,b,c,d the model parameters.

Based on biological consideration some refinements have been made and the IMAP modeling of the relationship is expressed by the equation:

$$P\left(t\right)=a\times \exp\left(\frac{b}{c}\left(1-\exp^{-ct}\right)\right)(1-\exp\left(d\left(t-e\right)\right)$$

with $P\left(t\right)$ the performance at age t et a,b,c,d,e the model parameters.

For each sex, both methods were applied to model the relation on age-specific best performances. The evolution of the relation over time was studied by applying this process to different periods: for men, data from 2001 to 2016 was firstly considered and then each next modeling adds one more year of data than the previous one, until reaching 2024. For women, the same process was applied, the only difference being that age-performance data was selected from 2006 onwards, since age data are missing until that date. The number of athletes for whom dates of birth have been recorded is 589 for men and 201 for women. The methodology (Berthelot et al., 2019) consisting of maximizing the coefficient of determination $R^{2}$ was applied to define each optimal parameter set. The adjusted coefficient of determination $R_{ajusted}^{2}$ was used to compare the two modelling. The coefficient of determination is considered *very weak* between 0 and 0.199, *weak* between 0.2 and 0.399, *medium* between 0.4 and 0.599, *strong* between 0.6 and 0.799 and *very strong* between 0.8 and 1.For a better convergence of the model, the performance data were centered and reduced according to the considered period then we added the minimum performance normalized so that all performance is strictly greater than 0. Age data was simply centered. Ages at peak performance, estimated peak performances and AUC (Area Under the Curve) on normalized predicted performances were computed for each considered period and sex to potentially exhibit changes that may have occurred over time. Linear regression was computed on these indicators to potentially reveal significantly changes over time. The differences in values between 2016 and 2021, 2016 and 2024, 2021 and 2024, as well as the minimum and maximum values, have been highlighted for peak ages in cases where its evolution demonstrates a statistically significant linear trend (p < 0.05).

## 3 Results

### 3.1 Skateboarding international competitive context evolution

**FIGURE 1:**
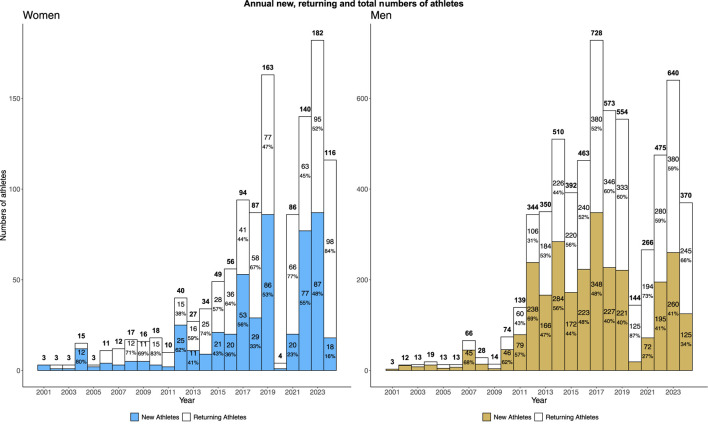
Annual percentages and numbers of total and new *versus* experienced athletes taking part in skateboarding international competitions.

Since 2016, the number of athletes taking part in international competitions has multiplied by 2.13 among men (1310 vs 2784 athletes) and by 4.02 among women (124 vs 498 athletes) reducing the ratio between the number of male and female athletes from 10.56 to 5.59 (Figure 1).

For male athletes, the record for the highest number of new competitors in a single year prior to 2016 was set in 2013, with 284 new athletes. Since, this record was surpassed once, in 2017, with a peak of 348 new male athletes (Figure 1). For female athletes, the highest number of new competitors prior to 2016 was 25, recorded in 2012. Since 2016, this record has been exceeded five times, in 2017, 2018, 2019, 2021, and 2023, with the highest number, 87 athletes, being set in 2023. (Figure 1).

For women, a low number of new athletes per year was observed from 2001 to 2011 (3.73 ± 3.07), while the number of new athletes per year tends to be higher during the period from 2012 to 2024 (35.15 ± 30.1). For men, the same trend is observed, although with different orders of magnitude, averaging 21.27 ± 24.55 new athletes per year from 2001 to 2011, compared to 196.15 ± 87.68 from 2012 to 2024. For both sex, Wilcoxon test indicated that 2001–2011 and 2012–2024 distributions of new athletes per year are significantly different.

### 3.2 Age-performance relationship

**FIGURE 2:**
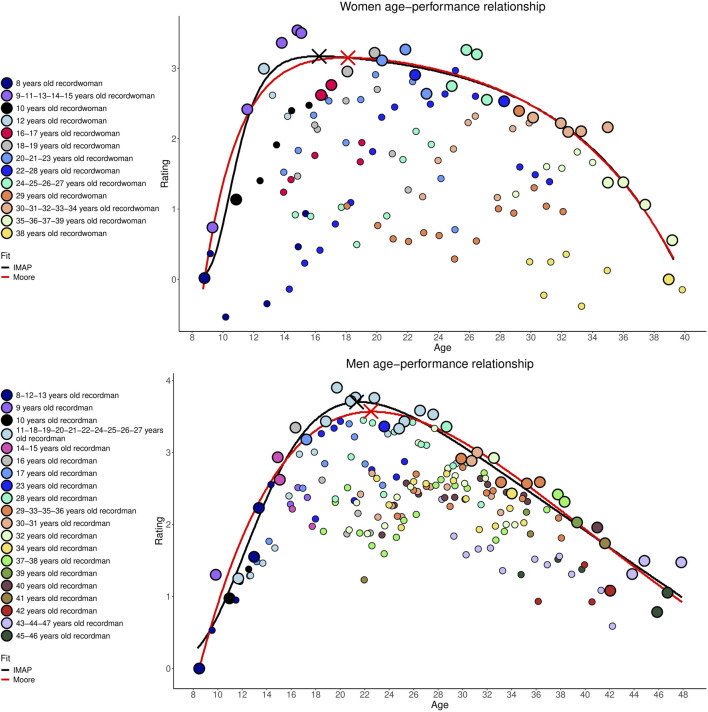
Men and women age-performance relationship models by Moore (red) and IMAP (black) using R with data from 2001 to 2024 Olympic Games. Two-point sizes are displayed. The larger points represent age-specific performance records for all athletes combined, used to fit the models, with each athlete who set an age-specific record assigned a different color. Some athletes have multiple age-specific performance records. The smaller points represent the age-specific performance records of individual athletes who have established a record at a given age. These smaller points are not included in the model fitting but serve to highlight the profiles and historical of athletes who have set these age-specific performance records.

For both men and women, the models fitted to the full data set provide a very strong explanation of the age-performance relationship ( $R^{2}$ between 0.9 and 0.94; Figure 2).

The Moore and IMAP models estimate that women reach their peak performance at ages 18.12 and 16.25 respectively. For men, the estimated peak ages are 22.51 and 21.34 respectively.

### 3.3 Age-performance relationship and its evolution over time

#### 3.3.1 Moore and IMAP performances

For women, Moore and IMAP models were able to strongly explain the age-performance relationship (R2 equal to 0.77 for both models) in 2016. Since 2017, this relationship is very strongly explained by the adjustment of these models (R2 by year between 0.87 and 0.91). For men, for all the adjustments made, the coefficients of determination by year are between 0.84 and 0.94, meaning that the Moore and IMAP models explain the age-performance relationship very strongly (Table 1).

**TABLE 1:** Estimated peak performances, peak ages and AUC evolution for men and women.

	*Women*						*Men*					
	*peak age*		*performance* *peak*		*AUC*		*peak age*		*performance peak*		*AUC*	
	*Moore*	*IMAP*	*Moore*	*IMAP*	*Moore*	*IMAP*	*Moore*	*IMAP*	*Moore*	*IMAP*	*Moore*	*IMAP*
2016	22.36	23.19	3.09	3.08	7.63	7.63	23.02	21.13	2.79	3.02	6.21	6.06
2017	23.92	23.92	3.32	3.41	7.99	8.02	22.44	20.5	2.75	2.96	6.02	5.89
2018	23.13	24.03	3.37	3.38	8.05	8.07	22.92	21.89	3.44	3.63	8.16	8.09
2019	21.35	22.24	3.52	3.65	9.03	9.11	23.16	22.11	3.32	3.58	7.88	7.82
2020	21.35	22.24	3.52	3.65	9.03	9.11	22.51	21.43	3.31	3.6	7.83	7.76
2021	19.63	20.53	3.43	3.41	8.91	8.91	22.5	21.43	3.4	3.62	8.08	8.02
2022	17.85	16.04	3.42	3.43	8.78	8.72	22.87	21.76	3.47	3.61	8.18	8.15
2023	18.12	16.25	3.15	3.17	7.8	7.73	22.5	21.33	3.55	3.69	8.31	8.28
2024	18.12	16.25	3.15	3.17	7.8	7.73	22.51	21.34	3.57	3.7	8.37	8.34
*Linear regression* *P-value*	0.0004 ***	0.0006 ***	0.853	0.7727	0.7708	0.9029	0.2439	0.5465	0.0046 **	0.0128 *	0.0099 **	0.0088 **
*Linear regression* $R^{2}$	0.8545	0.8358	0.005255	0.01271	0.01294	0.002282	0.1878	0.05423	0.7064	0.6116	0.6376	0.6483

#### 3.3.2 Peak performances, peak ages and AUC evolution

##### 3.3.2.1 Peak ages

For women, for both the Moore and IMAP models, the evolution of estimated peak ages over time follows a significant decreasing linear relationship (Table 1).

Table 2 shows the difference in years between the estimated peak ages for women at the beginnings and end of each considered period for both models. Last column displays the difference between maximum and minimum peak values recorded during the period 2016–2024.

**TABLE 2:** Peak age differences by period and maximum difference.

	2016–2021	2021–2024	2016–2024	Max-min
Moore	1.85	3.34	5.19	6.75
IMAP	2.68	3.34	6.02	7.08

Between 2021 and 2024, the age at peak decreased by 3.34 years according to both models, whereas between 2016 and 2021 the decrease fluctuated between 1.85 and 2.68. The maximum gaps between peak ages are 6.75 years for Moore (extremas: 2017 and 2022) and 7.08 years for IMAP (extremas: 2018 and 2022). The gaps measured between 2016 and 2024 are 5.19 and 6.02 years.

The evolution of the age at peak performance does not show any significant trend for men (Table 1).

##### 3.3.2.2 Peak performances and AUC

Peak performances and AUC evolution do not reveal any significant trend for women. For men, the increase in peak performances and AUC are linearly significant for both models (Table 1).

##### 3.3.2.3 Shape evolution

**FIGURE 3:**
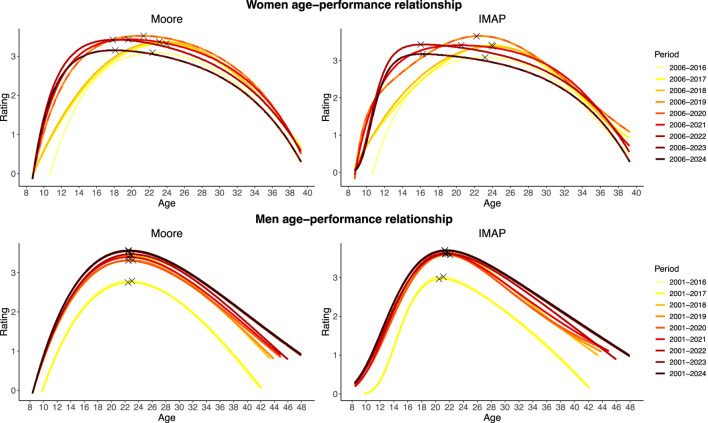
Moore and IMAP age-performances curves and peak performances from 2016 to 2024 Olympic Games.

Figure 3 enables to visualize the evolution of the shape of the performance distribution according to age between 2016 and 2024. The changes in performance trends for women reflect an improvement in the youth categories (elevation of the curve on its left segment). For Moore and IMAP, the performance levels estimated at ages 11 to 20 during the period 2006–2024 are higher than the performance levels estimated at these same ages during the period 2006–2016. The estimated performance levels stabilize at age 21. Subsequently, from ages 22 to 35 for Moore and 22 to 33 for IMAP, performance levels are higher during the 2006–2016 period. For men, the shape of the distribution does not differ between periods. Estimates of performance levels at each age are higher in the 2001–2024 period than in the 2016–2024 period.

## 4 Discussion

This study shows a decrease in the age at peak performance in female Street skateboard athletes as well as a widening of the optimal performance age zone in their male counterparts since the announcement of skateboarding’s inclusion in the Olympic program in 2016.

### 4.1 Expansion of career opportunities

Comparing the annual numbers of new athletes competing internationally since 2016 with the maxima previously recorded, corroborates Renfree et al. (2021) conclusions regarding the expansion of career opportunities offered by the inclusion of skateboarding in the Olympic Games. For male skateboarders, the past 8 years have seen as many new entrants into the international circuit as in the prior 15 years, with a peak in new participants in 2017, the year following skateboarding’s Olympic inclusion announcement. This effect is even more pronounced for female skateboarders, with over three times as many athletes beginning their international careers in the past 8 years compared to 2001–2016. Moreover, the annual record for new athletes set prior to 2016 has been surpassed 5 times over the 2016–2024 period, with a peak in 2023, aligning with community perceptions that Olympic inclusion has enabled female athletes to receive comparable support to their male counterparts, thus fostering skateboarding as a viable professional path (Renfree et al., 2021; D’Orazio, 2021). While gender parity in participation has not yet been fully achieved, the male-to-female participation ratio in international competitions since 2001 has strongly decreased, from 10.56 to 5.59 over the past 8 years. The difference between the average number of new athletes per year over the 2012–2024 period compared to the 2001–2011 period may suggest that skateboarding’s entry into the 2014 Youth Olympic Games program may also have played a role in the increase in the number of new athletes and the development of competition circuits.

### 4.2 Quantifying skateboarding performance

To explore the relationship between age and performance in skateboarding, a reliable quantification of performance is needed. Unlike disciplines such as track and field, swimming, or weightlifting, where performance is unequivocally measurable, skateboarding’s scoring systems are influenced by multiple subjective factors. Moreover, implementing consistent scoring protocols across competitions remains challenging, and existing data lacks the historical depth required for a robust longitudinal analysis of age-performance trends prior to skateboarding’s Olympic inclusion. However, the rating system employed in this study (Glickman and Hennessy, 2015) facilitates a quantifiable assessment of each athlete’s competitive level by accounting for past results and opponent quality. This system allows for consistent performance measurement across sports with end-of-competition rankings. Both the Moore and IMAP models demonstrate strong explanatory power, accounting for 90%–94% of performance variability among male and female street skateboarders over the studied period (2001–2024), with post-2017 values remaining above 86%.

### 4.3 Current age-performance relationship

Gender-based analysis reveals that female athletes currently achieve peak performance at a younger age than male athletes. This difference is estimated at 5.09 years by the IMAP model and 4.39 years by the Moore model, similar in magnitude to the 4.3-year gap observed between male and female artistic gymnasts at the London 2012 Olympics, the sport with the largest gendered age gap in peak performance (Longo et al., 2016). Female street skateboarders tend to reach their peak at younger ages compared to athletes across all Olympic sports in London 2012 (Longo et al., 2016). Their peak ages (18.12 years for Moore and 16.25 years for IMAP) are the closest to those seen in artistic gymnastics (19.4 years) and rhythmic gymnastics (21 years). Whereas for men, peak ages estimated respectively at 22.51 and 21.34 years according to Moore and IMAP are also the closest to sports classified in the group with the lowest peak ages: BMX cycling (23.2 years), diving (23.5 years), artistic gymnastics (23.7 years), weightlifting (24.5 years), pool swimming (24.6 years), boxing (24.8 years), middle distance athletics (25 years) (Longo et al., 2016).

### 4.4 Two Olympiads of age-performance relationship evolution

For male athletes, the peak age has remained stable, while the area under the curve (AUC) and peak performance have shown a linear increase. As rating is a variable that may experience inflation over time, age-specific performance records have been normalized relative to the periods studied. Thus, these increases do not necessarily indicate a rise in performance levels. Instead, it suggests a widening of the optimal performance age range, with more age-specific records approaching the all-ages performance record set in 2014. However, it remains essential to acknowledge that this trend is significantly influenced by a single athlete who continues to set age-specific performance records even after reaching the overall performance record. This highlights a limitation of population-based modeling and underscores the relevance of applying individual-level approaches, as suggested by Delarochelambert et al. (De Larochelambert et al., 2023). For women, the all-ages performance record continues to be broken regularly. Therefore the observed stability of AUC and peak performance over time indicates that the gap between the all-ages record and the age-specific records remains constant. The age zone of optimum performance is not becoming denser.

In contrast to their male counterparts, the age at peak performance for female athletes has shown a linear decline since 2016, with differences of 5.19 and 6.02 years between 2016 and 2024 according to Moore and IMAP. The extremas, recorded in 2017 and 2022 for Moore and in 2018 and 2022 for IMAP, reveal even larger differences of 6.75 and 7.08 years, reflecting increased participation following skateboarding’s Olympic inclusion, particularly among younger athletes (Renfree et al., 2021; D’Orazio, 2021). Due to the relatively low density of female competitors initially, new entrants have been able to set records soon after joining the circuit. Similar to the findings for men, the analysis of Figure 2 for women highlights the importance of considering the optimal performance age from an individual perspective (De Larochelambert et al., 2023) or according to career start age. Indeed, 3 of the 8 athletes who set age-specific performance records beyond Moore’s estimated peak age began their careers at an older age than the latter. The same applies to 4 out of 9 athletes for the IMAP peak age.

### 4.5 What about the future?

For women, the evolution over the coming years seems rather unpredictable. Indeed, on one hand, of the 6 athletes with age-specific performance records before Moore’s estimated peak age, 4 have not yet reached this estimated peak age. In the scenario where these athletes set new performance records in the coming years at ages above the estimated peak ages, the latter would tend to increase. On the other hand, the estimated peak age is 4 years higher for IMAP and 2 years higher for Moore than the all-ages performance record. If ever new age-specific performance records are set between the age of the all-ages record and the estimated peak ages, the latter could keep declining.

Moreover, as the pool of female athletes has been freshly renewed by the inclusion of skateboarding in the Olympic Games, consideration must be given to potential disparities in national skateboarding development policies and support for young athletes according to geographical area (Huebner and Perperoglou, 2019). Athletes from countries that are not currently leading in skateboarding may experience a more gradual progression, emphasizing structured learning and skill development as recommended in other disciplines (Boccia et al., 2017). This trajectory could lead them to achieve peak performance at later ages, thereby challenging the current trend toward early peak ages.

## 5 Conclusion

The inclusion of skateboarding in the Olympic Games program has led to an increase in international career opportunities, resulting in a decrease in the age at peak performance for female athletes and a widening of the optimal age range for male athletes. The characterization of the age-performance relationship established in this study is intended to enable skateboarding stakeholders to estimate which athletes are most likely to reach their best level at each of the next Olympiads, and to establish/adapt their young athlete development policy accordingly. Particular attention must be paid, however, to the evolution of age at peak performance of female athletes, whose future may not be predictable yet.

## Data Availability

The datasets used in this study are not publicly available, as they are provided by Gracenote to INSEP under a commercial collaboration. Consequently, they cannot be shared. However, the authors can provide details on the data structure and the transformations applied upon request.

## References

[B1] Aguilaniu B. Maitre J. Glenet S. Gegout-Petit A. Guenard H. (2008). European reference equations for CO and NO lung transfer. Eur. Respir. J. 31 (5), 1091–1097. 10.1183/09031936.00063207 18216061

[B2] Allen S. V. Hopkins W. G. (2015). Age of peak competitive performance of elite athletes: a systematic review. Sports Med. Auckl N. Z. 45 (10), 1431–1441. 10.1007/s40279-015-0354-3 26088954

[B3] Allen S. V. Vandenbogaerde T. J. Hopkins W. G. (2015). The performance effect of centralizing a nation’s elite swim program. Int. J. Sports Physiol. Perform. 10 (2), 198–203. 10.1123/ijspp.2014-0106 25010451

[B4] Berthelot G. Bar-Hen A. Marck A. Foulonneau V. Douady S. Noirez P. (2019). An integrative modeling approach to the age-performance relationship in mammals at the cellular scale. Sci. Rep. 23 janv 9 (1), 418. 10.1038/s41598-018-36707-3 PMC6344496 30674921

[B5] Berthelot G. Len S. Hellard P. Tafflet M. Guillaume M. Vollmer J. C. (2012). Exponential growth combined with exponential decline explains lifetime performance evolution in individual and human species. AGE. août 34 (4), 1001–1009. 10.1007/s11357-011-9274-9 PMC3682058 21695422

[B6] Berthelot G. Sedeaud A. Marck A. Antero-Jacquemin J. Schipman J. Saulière G. (2015). Has athletic performance reached its peak? Sports med auckl NZ. sept 45 (9), 1263–1271. 10.1007/s40279-015-0347-2 PMC4536275 26094000

[B7] Boccia G. Moisè P. Franceschi A. Trova F. Panero D. La Torre A. (2017). Career performance trajectories in track and field jumping events from youth to senior success: the importance of learning and development. PLOS ONE. 27 janv 12 (1), e0170744. 10.1371/journal.pone.0170744 PMC5271320 28129370

[B8] De Larochelambert Q. Barlier K. Hamri I. Difernand A. Sedeaud A. Toussaint J. F. (2023). Potential estimation model in French alpine skiing - individual evolution curve and progression typology. Front. Physiol. 13, 1082072. 10.3389/fphys.2022.1082072 36685191 PMC9849383

[B9] D’Orazio D. (2021). Skateboarding’s olympic moment: the gendered contours of sportification. J. Sport Soc. Issues 45 (5), 395–425. 10.1177/0193723520928595

[B10] Dormehl S. Robertson S. Williams C. (2016). Modelling the progression of male swimmers’ performances through adolescence. Sports 14 janv 4 (1), 2. 10.3390/sports4010002 PMC5968939 29910250

[B11] Glickman M. E. Hennessy J. (2015). A stochastic rank ordered logit model for rating multi-competitor games and sports. J. Quant. Anal. Sports 11(3), 131–144. 10.1515/jqas-2015-0012

[B12] Guillaume M. Len S. Tafflet M. Quinquis L. Montalvan B. Schaal K. (2011). Success and decline: top 10 tennis players follow a biphasic course. Med. Sci. Sports Exerc 43 (11), 2148–2154. 10.1249/MSS.0b013e31821eb533 21502889

[B13] Hollings S. C. Hopkins W. G. Hume P. A. (2014). Age at peak performance of successful track and field athletes. sept 9 (4), 651–661. 10.1260/1747-9541.9.4.651

[B14] Huebner M. Perperoglou A. (2019). Performance development from youth to senior and age of peak performance in olympic weightlifting. Front. Physiol. 10, 1121. 10.3389/fphys.2019.01121 31543826 PMC6732744

[B15] Knechtle B. Eichenberger R. Rosemann T. Lepers R. (2012a). Age and sex interactions in mountain ultramarathon running – the Swiss Alpine Marathon. Open Access J. Sports Med. juill 73, 73. 10.2147/oajsm.s33836 PMC3781902 24198590

[B16] Knechtle B. Rüst C. A. Knechtle P. Rosemann T. Lepers R. (2012c). Age-related changes in ultra-triathlon performances. déc 1 (1), 5. 10.1186/2046-7648-1-5 PMC3707100 23849327

[B17] Knechtle B. Rüst K. Rosemann T. Lepers R. (2012b). Age of peak performance in elite male and female Ironman triathletes competing in Ironman Switzerland, a qualifier for the Ironman world championship, Ironman Hawaii, from 1995 to 2011. Open Access J. Sports Med. 175, 175. 10.2147/oajsm.s37115 PMC3781912 24198600

[B18] Lara B. Salinero J. J. Del Coso J. (2014). The relationship between age and running time in elite marathoners is U-shaped. avr 36 (2), 1003–1008. 10.1007/s11357-013-9614-z PMC4039284 24407890

[B19] Lepers R. Cattagni T. (2012). Do older athletes reach limits in their performance during marathon running? AGE. juin 34 (3), 773–781. 10.1007/s11357-011-9271-z PMC3337940 21617894

[B20] Longo A. F. Siffredi C. R. Cardey M. L. Aquilino G. D. Lentini N. A. (2016). Age of peak performance in Olympic sports: a comparative research among disciplines. J. Hum. Sport Exerc 11, (1). 10.14198/jhse.2016.111.03

[B21] Marc A. Sedeaud A. Guillaume M. Rizk M. Schipman J. Antero-Jacquemin J. (2014). Marathon progress: demography, morphology and environment. J. Sports Sci. 3 (6):524–532. 10.1080/02640414.2013.835436 24191965

[B22] Marck A. Berthelot G. Foulonneau V. Marc A. Antero-Jacquemin J. Noirez P. (2017). Age-Related changes in locomotor performance reveal a similar pattern for *Caenorhabditis elegans*, Mus domesticus, *Canis familiaris*, *Equus caballus*, and *Homo sapiens* . J. Gerontol. A Biol. Sci. Med. Sci. 1 avr 72 (4), 455–463. 10.1093/gerona/glw136 PMC5861937 27522057

[B23] Millet G. P. Brocherie F. Burtscher J. (2021). Olympic sports science—bibliometric analysis of all summer and winter olympic sports research. Front. Sports Act. Living 3. Disponible sur. 10.3389/fspor.2021.772140 PMC8564375 34746779

[B24] Mitchell W. K. Williams J. Atherton P. Larvin M. Lund J. Narici M. (2012). Sarcopenia, dynapenia, and the impact of advancing age on human skeletal muscle size and strength; a quantitative review. Front. Physiol. 3. 260. 10.3389/fphys.2012.00260 22934016 PMC3429036

[B25] Moore D. H. (1975). A study of age group track and field records to relate age and running speed. Nat. janv 253 (5489), 264–265. 10.1038/253264a0 1113841

[B26] Norton K. Olds T. (2001). Morphological evolution of athletes over the 20th century: causes and consequences. Sports Med. Auckl N. Z. 31, 763–783. 10.2165/00007256-200131110-00001 11583103

[B27] Park D. C. Reuter-Lorenz P. (2009). The adaptive brain: aging and neurocognitive scaffolding. Annu. Rev. Psychol. 1 janv 60 (1), 173–196. 10.1146/annurev.psych.59.103006.093656 PMC3359129 19035823

[B28] Renfree G. Cueson D. Wood C. (2021). Skateboard, BMX freestyle, and sport climbing communities’ responses to their sports’ inclusion in the Olympic Games. Manag. Sport Leis. 24 29, 171–185. 10.1080/23750472.2021.2004211

[B29] Schulz R. Curnow C. (1988). Peak performance and age among superathletes: track and field, swimming, baseball, tennis, and golf. J. Gerontol. 1 Sept. 43 (5), P113–P120. 10.1093/geronj/43.5.p113 3418037

[B30] Simonton D. K. (1988). Age and outstanding achievement: what do we know after a century of research? Psychol. Bull. 104 (2), 251–267. 10.1037/0033-2909.104.2.251 3054997

[B31] Stanojevic S. Wade A. Stocks J. Hankinson J. Coates A. L. Pan H. (2008). Reference ranges for spirometry across all ages: a new approach. Am. J. Respir. Crit. Care Med. 1 févr 177 (3), 253–260. 10.1164/rccm.200708-1248OC PMC2643211 18006882

